# Moving toward the Light

**Published:** 1896-10

**Authors:** 


### Moving Toward the Light.

The long fight among the different schools of medicine has been based upon differences of opinion upon the so-called action of drugs; but intelligent physicians are finding out (many long ago made the discovery) that in the relation of the human body and drugs, it is the cells of the body which are active and not the drugs. The body acts upon the medicine, and not the medicine upon the body. Modern developments in hydrotherapy, electro-therapy, massotherapy, and the various branches of physiological medicine, including dietetics, have left comparatively little room for pharmaceutical products, so it is exceedingly foolish to still maintain the old quarrel about big doses and little doses, when doses of any sort have so small a part to play in the rational treatment of disease. The high-potency delusion seems about dead.—*Modern Medicine.*

